# Molecular Surveillance of *Cryptosporidium* spp., *Giardia duodenalis*, and *Enterocytozoon bieneusi* by Genotyping and Subtyping Parasites in Wastewater

**DOI:** 10.1371/journal.pntd.0001809

**Published:** 2012-09-06

**Authors:** Na Li, Lihua Xiao, Lin Wang, Shuming Zhao, Xukun Zhao, Liping Duan, Meijin Guo, Lili Liu, Yaoyu Feng

**Affiliations:** 1 State Key Laboratory of Bioreactor Engineering, School of Resources and Environmental Engineering, East China University of Science and Technology, Shanghai, People's Republic of China; 2 Division of Foodborne, Waterborne, and Environmental Diseases, National Center for Emerging and Zoonotic Infectious Diseases, Centers for Disease Control and Prevention, Atlanta, Georgia, United States of America; 3 Department of Blood Transfusion, Southwest Hospital, The Third Military Medical University, Chongqing, People's Republic of China; University of Washington, United States of America

## Abstract

**Background:**

Despite their wide occurrence, cryptosporidiosis and giardiasis are considered neglected diseases by the World Health Organization. The epidemiology of these diseases and microsporidiosis in humans in developing countries is poorly understood. The high concentration of pathogens in raw sewage makes the characterization of the transmission of these pathogens simple through the genotype and subtype analysis of a small number of samples.

**Methodology/Principal Findings:**

The distribution of genotypes and subtypes of *Cryptosporidium* spp., *Giardia duodenalis*, and *Enterocytozoon bieneusi* in 386 samples of combined sewer systems from Shanghai, Nanjing and Wuhan and the sewer system in Qingdao in China was determined using PCR-sequencing tools. *Eimeria* spp. were also genotyped to assess the contribution of domestic animals to *Cryptosporidium* spp., *G. duodenalis*, and *E. bieneusi* in wastewater. The high occurrence of *Cryptosporidium* spp. (56.2%), *G. duodenalis* (82.6%), *E. bieneusi* (87.6%), and *Eimeria*/*Cyclospora* (80.3%) made the source attribution possible. As expected, several human-pathogenic species/genotypes, including *Cryptosporidium hominis*, *Cryptosporidium meleagridis*, *G. duodenalis* sub-assemblage A-II, and *E. bieneusi* genotype D, were the dominant parasites in wastewater. In addition to humans, the common presence of *Cryptosporidium* spp. and *Eimeria* spp. from rodents indicated that rodents might have contributed to the occurrence of *E. bieneusi* genotype D in samples. Likewise, the finding of *Eimeria* spp. and *Cryptosporidium baileyi* from birds indicated that *C. meleagridis* might be of both human and bird origins.

**Conclusions/Significance:**

The distribution of *Cryptosporidium* species, *G. duodenalis* genotypes and subtypes, and *E. bieneusi* genotypes in urban wastewater indicates that anthroponotic transmission appeared to be important in epidemiology of cryptosporidiosis, giardiasis, and microsporidiosis in the study areas. The finding of different distributions of subtypes between Shanghai and Wuhan was indicative of possible differences in the source of *C. hominis* among different areas in China.

## Introduction

*Cryptosporidium*, *Giardia*, and microsporidia infections are significant causes of diarrhea in humans worldwide [1], [2]. Despite their wide occurrence, cryptosporidiosis and giardiasis are considered neglected diseases by the World Health Organization, largely due to a lack of studies in developing countries [3]. In addition to infecting humans, these parasites are found in a wide range of animals including domestic and wild animals. Humans can acquire *Cryptosporidium* infections through several transmission routes, such as direct contact with infected persons (anthroponotic transmission) or animals (zoonotic transmission) and ingestion of contaminated water or food [4]. Although the epidemiology of *Giardia* and microsporidia is less clear, potential sources of infections can be identified by comparing the distribution of genotypes of each parasite among different hosts [5], [6].

The host-adaptive nature of different species/genotypes of these parasites helps us understand the potential infection sources and transmission routes of the disease. Among the over 70 species and genotype of *Cryptosporidium*, five species are responsible for most human infections, including *C. hominis* infecting humans, *C. parvum* infecting ruminants, *C. meleagridis* infecting birds, *C. felis* infecting cats, and *C. canis* infecting dogs [7]. In contrast, giardiasis in humans and most other mammals is caused by *Giardia duodenalis* (also known as *Giardia lamblia* or *Giardia intestinalis*) [6]. *Giardia duodenalis* has at least eight genotypes, assemblages A-H. Among them, assemblages A and B infect humans and a broad range of other hosts, including livestock, cats, dogs, and wild mammals. Within the assemblage A, there are three major subtypes groups (sub-assemblages), A-I, A-II and A-III, with A-I mainly infecting most animals, A-II mainly infecting humans, and A-III mainly infecting wild ruminants. Assemblages C-H appear generally to be mostly restricted to companion animals, livestock, rodents, and seals [6]. *Enterocytozoon bieneusi* is the most common microsporidian species infecting humans, domestic animals, wild mammals, and birds [5], [8]. *Enterocytozoon bieneusi* forms several phylogenetic groups of genotypes, with group 1 infecting humans and most animals and groups 2–5 infecting different animals [1], [9]. Groups 2–5 represent host-adapted genotypes that have no major public health significance [10], [11]. Therefore, using both an understanding of the host-specificity of various pathogen species/subtypes and describing the most common pathogen species/subtypes in wastewater can shed light into the potential infections sources and transmission routes of diseases. For example, a common occurrence of the human-specific *C. hominis* rather than potentially zoonotic *C. parvum* would indicate that anthroponotic transmission is important in cryptosporidiosis transmission.

The monitoring of raw wastewater for pathogens has been used in surveillance of a few bacterial, viral and parasitic pathogens in urban communities [12]–[16]. The identification of pathogens in human specimens is still the standard method for the elucidation of disease transmission. However, because of the low prevalence of most pathogens, the screening of a large number of specimens is frequently needed, which is time-consuming and expensive. In contrast, the high concentration of pathogens in raw sewage facilities the detection of these pathogens via the analysis of a small number of samples and can provide a quick overall picture of the disease transmission at the community level, although some zoonotic pathogens in wastewater may also come from domestic animals. Therefore, this approach is especially useful for emerging infectious pathogens, such as *E. coli* O157:H7 and H5N1. Because some emerging infectious diseases are also endemic in urban communities in addition to causing large-scale outbreaks of illnesses, simple detection of the pathogens in raw wastewater is not enough for molecular surveillance of the pathogens. For these endemic pathogens, molecular epidemiologic tracking (subtyping) is frequently needed to assess the endemic transmission at the community level and to identify the occurrence of outbreaks, which are characterized by the presence of one or a few subtypes at high frequency.

In two U.S. studies, genotyping and subtyping parasites in raw wastewater was used as a rapid and cost-effective tool for characterizing the transmission of cryptosporidiosis and giardiasis in an urban community [15], [17]. In one study, promising results were obtained in molecular surveillance of an outbreak strain of *C. hominis* in urban wastewater [15]. Our previous study on species and subtype distribution of *Cryptosporidium* spp. in domestic wastewater in Shanghai, China suggested that anthroponotic transmission might play an important role in cryptosporidiosis epidemiology, and the dominant *C. hominis* subtypes in this region were different from those in other countries [16]. The objectives of this study were to identify the species/genotype distribution of *Cryptosporidium* spp., *G. duodenalis*, and *E. bieneusi* in domestic wastewater in four large cities in China, to assess endemic transmission of cryptosporidiosis, giardiasis and microsporidiosis based on the species or genotype identified and our knowledge on their host sources and geographic distribution, and to determine whether the unique subtypes of *C. hominis* identified in Shanghai also existed in other areas in China. Because domestic animals may contribute to parasite occurrence in urban wastewater, *Eimeria* spp., which commonly infect domestic mammals and birds and have strong host specificity, were also analyzed to facilitate the animal source attribution of *Cryptosporidium*, *G. duodenalis,* and *E. bieneusi* genotypes found in wastewater.

## Materials and Methods

### Wastewater sample collection and processing

A total of 386 raw wastewater samples were collected from four cities in China from 2006 to 2009, including 90 from four wastewater treatment plants (WWTPs) in Shanghai (December 2006 to April 2007), 87 from five WWTPs in Nanjing (July 2007 to May 2009), 109 from five WWTPs in Qingdao (April–July 2008), and 100 from five WWTPs in Wuhan (April–July 2008). All WWTPs in each city were located at areas with high population density. The four cities were selected based on the following criteria: (1) different population densities, with 3,631 people/km^2^ in Shanghai, which is the highest in mainland China, and 815 to 890 people/km^2^ in the other three cities; (2) different pet densities, with the highest in Shanghai; (3) different conditions of the sewage system, with the best in Shanghai and more antique in Qingdao. Qingdao has separate storm water and domestic wastewater systems, and the other three cities use combined sewer systems in the inner city areas. Grab samples of 500 to 1,000 ml of raw wastewater were collected at the input of WWTPs. Cysts, oocysts and spores of parasites in samples were concentrated by centrifugation at 6,000×g for 10 min. Samples were stored in 2.5% potassium dichromate solution at 4°C prior to DNA extraction.

### DNA extraction

After washing the samples twice in distilled water, genomic DNA was extracted from 0.5 ml of concentrate using a FastDNA SPIN Kit for Soil (BIO 101, Carlsbad, CA) and eluted in 100 µl of reagent-grade water as described previously [18]. DNA was stored at −80°C until analyzed by PCR. DNA from each sample was analyzed at least five times for each genetic target using 2 µl of the extraction DNA in PCR. Following analysis with a nested PCR described below, secondary PCR products were detected by gel electrophoresis in 1.5% agarose.

### *Cryptosporidium* detection, genotyping and subtyping

A ∼830-bp fragment of the small subunit (SSU) rRNA gene was amplified by nested PCR as previously described [19]. *Cryptosporidium* species and genotypes were differentiated by restriction fragment length polymorphism (RFLP) analysis of the secondary PCR products using restriction enzymes *Ssp*I and *Vsp*I [20]. *Cryptosporidium muris* and *C. andersoni* were further differentiated by RFLP analysis using restriction enzymes *Dde*I [21]. To identify *C. parvum*, *C. hominis* and *C. cuniculus* subtypes, a ∼850-bp fragment of the 60 kDa glycoprotein (gp60) gene was amplified by nested PCR [16]. The secondary PCR products were sequenced using the secondary PCR primers and an intermediary sequencing primer (5′-GAGATATATCTTGTTGCG-3′). The established subtype nomenclature [22] was used to classify gp60 subtypes.

### *Giardia duodenalis* detection, genotyping and subtyping

To detect *G. duodenalis*, a 530-bp fragment of the triosephosphate isomerase (tpi) gene was amplified by nested PCR [23]. Genotypes and subtypes of *G. duodenalis* were determined by sequence analysis of the secondary PCR products.

### *Enterocytozoon bieneusi* detection and genotyping

To detect *E. bieneusi*, a ∼392-bp fragment of the ribosomal internal transcribed spacer (ITS) was amplified by nested PCR [10]. Genotypes of *E. bieneusi* were determined by sequence analysis of the secondary PCR products, and named according to the established nomenclature [24].

### Detection and identification of *Eimeria* spp. and *Cyclospora cayetanensis*

To detect *Eimeria* spp. and *Cyclospora* spp., a 294-bp fragment of the SSU rRNA gene was amplified by nested PCR [25]. The differentiation of *Eimeria* and *Cyclospora* species were done by sequence analysis of the secondary PCR products.

### DNA sequencing and data analysis

After being purified using the Montage PCR filters (Millipore, Bedford, MA), the secondary PCR products were sequenced directly with the secondary PCR primers using an ABI BigDye Terminator v. 3.1 cycle sequencing kit (Applied Biosystems, Foster City, CA) and the manufacturer-suggested procedures. Sequences were read on an ABI3130 Genetic Analyzer (Applied Biosystems). To confirm the accuracy of the sequence and to detect multiple genotypes and subtypes in a sample, all positive PCR products from each sample were sequenced, except for *C. muris*-positive samples in Qingdao, which was identified mostly by *Ssp*I and *Dde*I RFLP analysis, and for *Eimeria*/*Cyclospora*-postive samples, for which only one PCR product per sample was sequenced. The nucleotide sequences of *Cryptosporidium* spp., *G. duodenalis*, *E. bieneusi*, and *Eimeria/Cyclospora* spp. were aligned with reference sequences downloaded from the GenBank using ClustalX (ftp://ftp-igbmc.u-strasbg.fr/pub/ClustalX) to determine their genotype or subtype identity. Each pathogen genotype or subtype was presented as the number of positive samples or PCR products, with its frequency calculated based on the total number of samples analyzed in each area.

### Phylogenetic analysis

The genotypes of *Enterocytozoon bieneusi* obtained were compared to known *E. bieneusi* genotypes by phylogenetic analysis of ITS sequences. After eliminating sequences with ambiguous positions, 579 *E. bieneusi* ITS sequences were obtained from wastewater samples, representing 23 genotypes. They were used in a neighbour-joining analysis together with reference sequences downloaded from the GenBank, using 500 replicates in bootstrap assessment of the robustness of clusters. Likewise, 298 clean SSU rRNA sequences of *Eimeria/Cyclospora* representing 39 genotypes were assigned to host groups based on their phylogenetic positions in a similarly constructed neighbour-joining tree with reference sequences from known animal hosts.

### Nucleotide sequence accession numbers

Unique nucleotide sequences generated from the study were deposited in GenBank under accession numbers JQ863254-JQ863307.

## Results

### *Cryptosporidium* species/genotypes in wastewater

Based on PCR analysis of the SSU rRNA gene, 63 of 90 (70.0%) wastewater samples from Shanghai, 32 of 87 (36.8%) samples from Nanjing, 75 of 109 (68.8%) samples from Qingdao, and 47 of 100 (47.0%) samples from Wuhan were positive for *Cryptosporidium* spp. RFLP analysis and DNA sequencing of PCR products revealed the presence of 14 species/genotypes of *Cryptosporidium* in these samples: *C. hominis*, *C. parvum*, *C. andersoni, C. canis, C. suis, C. cuniculus*, *C. meleagridis*, *C. baileyi, C. muris*, pig genotype II, Avian genotype III, rat genotype I, rat genotype IV, and a new genotype (Table 1).

**Table 1:** *Cryptosporidium* species and genotypes in wastewater samples from four cities in China during 2006–2009.

Species/genotype or gp60 subtype	Ref No.	No. of positive samples (No. of positive PCR products)^a^			
		Shanghai^b^	Nanjing	Qingdao	Wuhan
Humans					
*C. hominis*	EF570922 or AY204230	59 (78)	6 (9)	1 (1)	19 (21)
IaA18R4	FJ153246	3 (3)			
IaA19R4	FJ153245	3 (3)			
IaA27R3	AF164502		1 (1)		
IbA19G2	FJ707312	5 (6)^c^	1 (1)		
IbA20G2	FJ707313	3 (3)			
IbA21G2	FJ153239	22 (35)^c^			
IdA14	GU214350	2 (2)			
IdA16	HQ149034	2 (3)	1 (1)		1 (1)^d^
IeA11G3T3	GU214354				1 (1)
IeA12G3T3	FJ153242	6 (7)	1 (1)	1 (1)	9 (12)^d^
IfA20G1	FJ153243	1 (1)			
IfA22G1	FJ153244	4 (5)			
*Cryptosporidium* W13314	HM191258	1 (1)			
Livestock and companion animals					
*C. parvum*	AF093490	1 (1)		2 (3)	
IIdA19G1	HQ009809			2 (3)	
*C. andersoni*	FJ463187				4 (5)
*C. canis*	EU754833			1 (2)	1 (1)
*C. suis*	AF115377 or GQ345008	1 (2)	7 (7)	6 (7)	17 (20)
Pig genotype II	AB449825		1 (1)		5 (5)
*C. cuniculus*	EU437413		3 (3)		2 (2)
VaA31	FJ262734		1 (1)		
Birds					
*C. meleagridis*	AF112574	7 (7)	2 (2)	7 (7)	6 (6)
*C. baileyi*	AF093495 or EU717825	3 (3)	6 (6)	2 (2)	4 (4)
Avian genotype III	HM116386	1 (1)			
Rodents					
*C. muris*	AF093498	1 (1)	18 (24)	2 (2)^e^	10 (10)
Rat genotype I	AY268584	1 (1)	1 (1)	2 (2)	1 (2)
Rat genotype IV	AY737585, AY737580 or AY737584			8 (8)	2 (2)
Total^f^		63/90 (70.0%)	32/87 (36.8%)	75/109 (68.8%)	47/100 (47.0%)

a All data on specific genotypes and subtypes are based on sequence analysis.

b *Cryptosporidium* data from Shanghai wastewater samples were previously reported in [16].

c Concurrent presence of IbA19G2 and IbA21G2 was found in one sample.

d Concurrent presence of IdA16 and IeA12G3T3 was found in one sample.

e A total of 60 (109) were positive for *C. muris by Ssp*I and *Dde*I RFLP analysis.

f No. of positive samples/No. of samples (positivity in percentage) based on PCR analysis of the SSU rRNA gene.

Among the five major human-pathogenic *Cryptosporidium* species (*C. hominis*, *C. parvum*, *C. meleagridis*, *C. canis*, and *C. felis*), both the human-specific *C. hominis* and bird-adapted *C. meleagridis* were found in samples from all four cities. The occurrence of *C. hominis* was more common than *C. meleagridis* in Shanghai (59 versus seven), Nanjing (six versus two), and Wuhan (19 versus six). The only exception was Qingdao where *C. hominis* was found in only one sample while *C. meleagridis* was found in seven samples (Table 1). Similar to the common existence of *C. meleagridis* (22 samples), other species from birds, such as *C. baileyi* was also detected in 15 samples from the four cities studied. *Cryptosporidium parvum*, which is a common parasite of cattle, was found only in one sample in Shanghai and two samples in Qingdao. The rare occurrence of *C. parvum* was consistent with the frequency of other species infecting cattle, such as *C. andersoni* (found in four samples only). *Cryptosporidium canis*, commonly found dogs, was only seen in one sample each in Qingdao and Wuhan, where the common species in cats, *C. felis*, was not detected in any of the samples.

Surprisingly, *C. muris* and other *Cryptosporidium* genotypes infecting rodents were the most commonly detected species/genotypes in Qingdao, being found in 70 samples, or 93.3% of all *Cryptosporidium*-positive samples. Within *C. muris* from Qingdao, the detection in 12 samples was confirmed by DNA sequencing and the other 58 diagnosed by RFLP analysis with restriction enzymes *Ssp*I and *Dde*I (Table 1). Although these species/genotypes were also found at a high frequency in Nanjing (19 samples, or 59.4% of *Cryptosporidium*-positive samples), they were less commonly seen in Wuhan (13 or 27.7% *Cryptosporidium*-positive samples), and were only rarely seen in Shanghai (two samples, or 3.2% *Cryptosporidium*-positive samples) (Table 1).

The partial SSU rRNA gene sequence of a novel genotype in this study (FJ153238) was identical to that isolated from a patient in the United Kingdom (HM191258) [26]. A recently identified zoonotic species, *C. cuniculus* [27], which was responsible for a waterborne outbreak in the United Kingdom in 2008 [28], was also found in five samples in this study. The concurrent presence of multiple *Cryptosporidium* species/genotypes was seen in many samples: two species/genotypes in 44 samples, three species/genotypes in 13 samples, and four species/genotypes in six samples.

### *Cryptosporidium hominis*, *C. parvum* and *C. cuniculus* gp60 subtypes

Samples positive for *C. hominis*, *C. parvum* or *C. cuniculus* were subtyped by sequence analysis of the gp60 gene. For *C. hominis*-positive samples, 47 of 59 samples in Shanghai, four of six samples in Nanjing, one sample in Qingdao, and 10 of 19 samples in Wuhan were successfully subtyped. Altogether, there were 12 subtypes in five subtype families in the 62 *C. hominis*-positive wastewater samples: Ia (three subtypes in seven samples), Ib (three subtypes in 30 samples), Id (two subtypes in six samples), Ie (two subtypes in 18 samples), and If (two subtypes in five samples) (Table 1). Although Ib subtypes were commonly detected in Shanghai samples (30/48 *C. hominis*-positive samples subtyped successfully), only one sample in Nanjing was positive for one of the Ib subtypes (IbA19G2). In contrast, the Ie subtype IeA12G3T3 that previously thought to be unique to Shanghai, was found in 11 samples in the other three cities. Of the five *C. cuniculus*-positive samples in Nanjing and Wuhan, only one was successfully subtyped as VaA31 (Table 1). Two of the three *C. parvum*-positive samples were subtyped as IIdA19G1 (Table 1). Concurrent presence of two subtypes was found in five samples, including IbA19G2 and IbA21G2 in one sample from Shanghai, and IdA16 and IeA12G3T3 in one sample from Wuhan. Except for samples from Qingdao, all *C. meleagridis*-positive samples were also analyzed by gp60 PCR. Only four of the seven samples from Shanghai and one of the six samples from Wuhan produced the expected gp60 PCR products, all of which belonged to *C. hominis* subtypes indicating the concurrent presence of *C. hominis*.

### *Giardia duodenalis* genotypes and subtypes

The PCR analysis of tpi gene identified a high occurrence of *G. duodenalis* in the four cities studied, with positive rates of 67.8% (59/87) in Nanjing, 69.0% (69/100) in Wuhan, 94.5% (85/90) in Shanghai and 97.2% in Qingdao (106/109). All of them belonged to two known genotypes of *G. duodenalis* (assemblages A and B) of humans. The prevalent genotype was assemblage A, which was detected in 76.1% (243 of 319) *G. duodenalis*-positive samples, including 51 samples in Shanghai, 38 samples in Nanjing, 97 samples in Qingdao, and 57 samples in Wuhan (Table 2). Assemblage B was found in 59 samples, but in 53 of them it was in concurrence with assemblage A (Table 2). In 27 samples, the presence of mixed genotypes was identified by the occurrence different assemblages in different replicates of analysis while in the remaining 36 samples the presence of mixed genotypes was demonstrated by underlying peaks in DNA sequencing trace files. All samples containing assemblage A had the A2 subtype. Sixteen assemblage B subtypes were found in this study, only one of which was identical to a sequence reported before (GenBank No. L02116).

**Table 2:** *Giardia duodenalis* assemblages and genotypes in wastewater samples of four cities in China during 2006–2009.

Genotype or subtype	Ref No.	No. of positive samples (No. of positive PCR products)^a^			
		Shanghai	Nanjing	Qingdao	Wuhan
Humans					
A2	U57897	51 (105)	38 (76)	97 (180)	57 (86)
Mammals (including humans)					
B1	L02116	1 (1)			
B2	17238-1			1 (1)	
B3	17257-2		1 (1)		
B4	17239-1		1 (1)		
B5	19068-2			1 (1)	
A2+B1^b^	U57897, L02116	1 (1)			
A2+B2^b^	17238-1		2 (5)		
A2+B3^b^	17257-2		3 (6)		
A2+B4^b^	17239-1		1 (2)		
A2+B6^b^	17219-2		1 (2)		
A2+B7^b^	18930-2				1 (2)
A2+B8^b^	17231-2		1 (5)		
A2+B9^b^	18933-2				1 (2)
A2+B10^b^	17252-1		1 (4)		
A2+B11^b^	18972-2				1 (2)
A2+B12^b^	17259-1		1 (2)		
A2+B13^b^	19088-1			1 (2)	
A2+B14^b^	17241-2		1 (2)		
A2+B15^b^	19121-2			1 (2)	
A2+B^c^		30 (38)	6 (10)		
B2+B16	17238-1, 19108-2			1 (2)	
Total^d^		85/90 (94.5%)	59/87 (67.8%)	106/109 (97.2%)	69/100 (69.0%)

a All data on specific genotypes and subtypes are based on sequence analysis.

b Different sequencing results in different PCR replicate analyses of each sample. B2–B16 are new subtypes identified in this study.

c Based on the occurrence of underlying peaks in DNA sequencing trace files; the subtype identity of assemblage B could not be established under such a circumstance.

d No. of positive samples/No. of samples (positivity in percentage) based on PCR analysis of the tpi gene.

### *Enterocytozoon bieneusi* genotypes

*Enterocytozoon bieneusi* was detected in more than 90% of samples in Shanghai, Qingdao and Wuhan, with prevalence lower in Nanjing (62.1% or 54/87 samples). In the 338 *E. bieneusi*-positive samples, a total of 23 ITS genotypes were seen in 579 sequences obtained (Table 3). Genotype D was the most prevalent, being found in 279 of 338 (82.5%) *E. bieneusi*-positive samples or 469 of 592 (79.2%) sequences.

**Table 3:** *Enterocytozoon bieneusi* ITS genotypes in wastewater samples of four cities in China during 2006–2009^a^.

Genotype	Ref No.	Reported hosts in the literature	No. of positive samples (No. of positive PCR products)^a^			
			Shanghai	Nanjing	Qingdao	Wuhan
Humans and/or animals	Group 1a–1h^b^ (Group I)^c^					
Type IV	AY371277	Humans, cats, cattle and dogs	4 (4)		5 (5)	3 (3)
EbpC	AY371279	Humans, pigs, beaver, otter, muskrat, raccon and fox	2 (2)		1 (1)	5 (5)
EbpD	AF076043	Pigs			1 (1)	
Peru6	AY371281	Humans, dog, cattle, birds		1 (1)	3 (4)	1(1)
Peru8	AY371283	Humans	1 (1)	1 (1)		11 (13)
Peru11	AY371286	Humans	2 (2)	2 (2)		6 (6)
C	AF101199	Humans				1 (1)
D	AY371284	Humans, pigs, cattle, dogs, beaver, falcons, fox, macaque, muskrat, raccoon	78 (138)	50 (99)	71 (99)	80 (133)
EbpA	AF076040	Cattle, pigs				2 (2)
PtEb IV	DQ885580	Cats			1 (1)	
BEB6	EU153584	Cattle			1 (1)	1 (1)
PigEBITS7	AF348475	Pigs	6 (7)		1 (1)	
PigEBITS8	AF348476	Pigs	1 (1)			
WL12	AY237220	Beaver, otter		1 (1)		
WL14	AY237222	Muskrat		2 (2)		
Unknown	Group 1i^d^					
WW1^e^	19031-2				4 (5)	
WW2^e^	19055-2				1 (1)	
WW3^e^	19083-1				15 (17)	5 (6)
WW4^e^	19063-2				3 (3)	
WW5^e^	19054-1				1 (1)	
Muskrat and cat	Group 3^b^ (Group I^c^)					
WL4	AY237212	Muskrat			1 (1)	1 (1)
Unknown	Group 6^d^					
WW6^e^	19081-2				4 (4)	
WW7^e^	19107-1				1 (1)	
Dog	Outliers^b^ (Group IV^c^)					
PtEb IX	DQ885585	Dogs			4 (4)	3 (3)
WW8^e^	19029-1				4 (5)	
WW9^e^	19079-1				1 (1)	
Total^f^			85/90 (94.5%)	54/87 (62.1%)	100/109 (91.7%)	99/100 (99.0%)

a All data on specific genotypes are based on sequence analysis.

b Group name given by Thellier and Breton (2008).

c Group name given by Henriques-Gil et al. (2010).

d Group or subgroup newly identified in this study.

e New genotype in this study.

f No. of positive samples/No. of samples (positivity in percentage) based on PCR analysis of the ITS.

**Figure 1:**
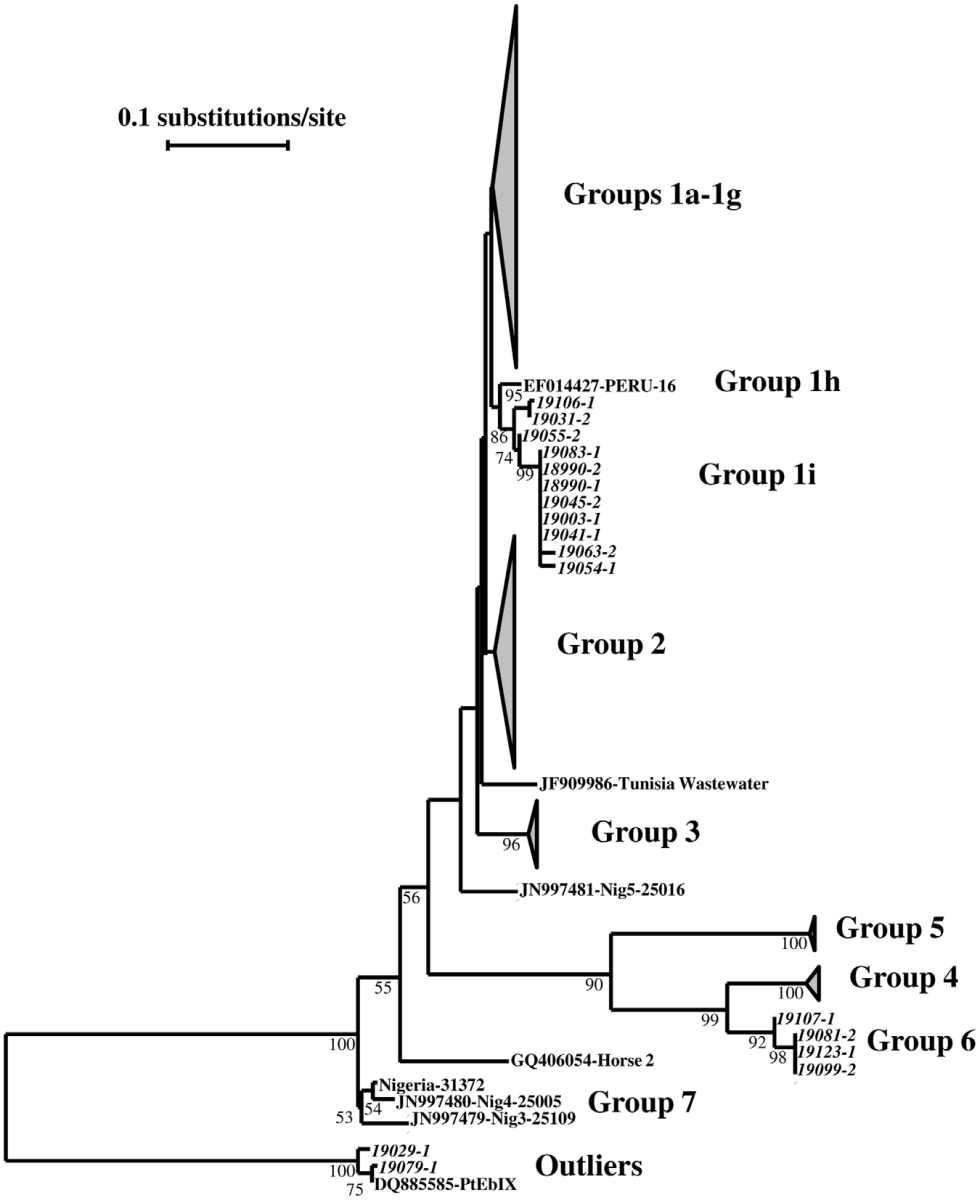
Phylogenetic relationship of *Enterocytozoon bieneusi* genotype groups. The relationship of new *E. bieneusi* genotype groups (1i and 6) identified in this study to established *E. bieneusi* genotype groups was inferred by a neighbor-joining analysis of nucleotide sequences of the ITS, based on Kimura 2-parameter distances. Some sequences generated in the study are italicized, while the remaining 538 sequences in Groups 1a–1g and one sequence in Group 3 are not shown (see Table 3). Several unique ITS sequences from AIDS patients in a recent Nigerian study [43] are designed as Group 7.

Two new groups of *E. bieneusi* genotypes were found in this study. A new group named 1i consisted of five genotypes seen in 33 sequences and formed a cluster sister to the previously designed subgroup 1 h [9]. This cluster was within the large group (group 1) of ITS sequences originated from humans and animals [1], [9], [29]. The other new group was named as group 6 in this study and consisted of two genotypes seen in five sequences (Fig. 1). In addition, we also found two new genotypes belonged to the so called Outliers group from dogs [1]; one was seen in five sequences and the other one in one sequence. Consistent with the high prevalence of *E. bieneusi*, 34 samples had concurrent presence of two genotypes.

### *Eimeria* spp. and *Cyclospora cayetanensis*

Among 386 samples analyzed, 310 samples (80.3%) were positive for *Eimeria* spp. or *Cyclospora* spp. The majority of the PCR products were from *Eimeria* spp., such as *Eimeria alabamensis* and its relative from cattle (167 samples), *E. acervulina, E. tenella* and their relatives from chicken (six samples), *E. nieschulzi, E. falciformis, E. separata* and their relatives from rodents (95 samples), *E. flavecens* from rabbits (one sample), and *E. arnyi* relatives from snakes (four samples). *Cyclospora* spp. were detected in 13 wastewater samples; in two samples, the sequence obtained had minor differences from *C. cayetanensis*. Nine *Eimeria* spp. of unknown animal sources were also found in 13 sequences from 13 samples in this study (Table 4).

**Table 4:** *Eimeria* species and related parasites in wastewater samples of four cities in China during 2006–2009^a^.

Species or genotype	Ref No.	No. of positive samples (No. of positive PCR products)^a^			
		Shanghai	Nanjing	Qingdao	Wuhan
*Eimeria* spp. from cattle (?)					
*E. alabamensis*	AF291427	1 (1)	12 (12)	38 (38)	43 (43)
*Eimeria* sp.	18097	1 (1)	7 (7)	27 (27)	37 (37)
*Eimeria* sp.	22035		1 (1)		
*Eimeria* spp. from chicken					
*E. acervulina*	EF210324		1 (1)		
*E. tenella*	U40264		1 (1)		
*Eimeria* sp.	26221		2 (2)		
*Eimeria* sp.	17236		1 (1)		
*Eimeria* sp.	17242		1 (1)		
*Eimeria* spp. from rabbits					
*E. flavescens*	EF694011		1 (1)		
*Eimeria* spp. from snakes					
*Eimeria* sp.	18110	2 (2)			
*Eimeria* sp.	18118	1 (1)			
*Eimeria* sp.	22024		1 (1)		
*Eimeria* spp. from rodents					
*E. nieschulzi*	U40263	4 (4)	8 (8)	11 (11)	
*E. separata*	AF311643	5 (5)	6 (6)	2 (2)	1 (1)
*Eimeria* sp.	AB447983		1 (1)	5 (5)	2 (2)
*E. falciformis*	AF080614	1 (1)	1 (1)		
*Eimeria* sp.	19027	6 (6)	3 (3)	13 (13)	4 (4)
*Eimeria* sp.	22032		3 (3)		4 (4)
*Eimeria* sp.	19118	3 (3)		1 (1)	
*Eimeria* sp.	19110			2 (2)	1 (1)
*Eimeria* sp.	19108			2 (2)	
*Eimeria* sp.	17256		1 (1)		
*Eimeria* sp.	22029		1 (1)		
*Eimeria* sp.	19111			1 (1)	
*Eimeria* sp.	18928				1 (1)
*Eimeria* sp.	18969				1 (1)
*Eimeria* sp.	18934				1 (1)
*Eimeria* spp. from unknown source					
*Eimeria* sp.	18109	3 (3)			
*Eimeria* sp.	18132	2 (2)			
*Eimeria* sp.	18079	2 (2)			
*Eimeria* sp.	18121	1 (1)			
*Eimeria* sp.	18076	1 (1)			
*Eimeria* sp.	18120	1 (1)			
*Eimeria* sp.	18137	1 (1)			
*Eimeria* sp.	18075	1 (1)			
*Eimeria* sp.	18100	1 (1)			
*Cyclospora* spp. from humans and primates					
*C. cayetanensis*	AF111183		5 (5)	6 (6)	
*Cyclospora* sp.	26218		1 (1)		
*Cyclospora* sp.	18979				1 (1)
Total^b^		42/90 (46.7%)	60/87 (69.0%)	109/109 (100%)	99/100 (99.0%)

a All data on specific species/genotypes are based on sequence analysis.

b No. of positive samples/No. of samples (positivity in percentage) based on PCR analysis of the SSU rRNA gene.

## Discussion

The transmission of *Cryptosporidium*, *G. duodenalis* and *E. bieneusi* in humans in China is poorly understood. There are only three studies that have genotyped and subtyped *Cryptosporidium* spp. in humans. All five specimens collected from diarrheic children in Tianjin had *C. hominis* [30]. Nine specimens were positive for *C. hominis* and one for *C. felis* in a small study of out-patients in Henan Province [31]. In a study conducted in in-patients in three pediatric hospitals in Shanghai, *C. hominis* was identified in 92, *C. meleagridis* in 6, *C. felis* in 2, and *C. canis* in 2 cases, but the majority of *C. hominis*-positive patients (74/92) were from a hospital-associated outbreak of cryptosporidiosis, which had made less samples available for the examination of transmission dynamics in the general population. Likewise, there are only three small-scale studies of *G. duodenalis* genotypes in humans in China. In one study in Anhui Province, assemblages A and B were each found in four patients [32]. In another study in Hebei Province, sub-assemblage A-II was found in three patients [33]. In a recent study in Henan Province, sub-assemblages A-I and A-II and assemblage B were was found in eight, four and six patients, respectively [31]. In the only study on *E. bieneusi* infection in China, genotypes I and J and four novel genotypes were found in nine diarrheic children in Jilin Province [34].

Data generated in this study suggest that anthroponotic transmission is important in the epidemiology of cryptosporidiosis in the study areas. This was supported by the common finding of *C. hominis* in the wastewater samples in Shanghai and Wuhan, where 93.7% and 40.4% of *Cryptosporidium*-positive samples had *C. hominis*, respectively. The proportion of samples positive for *C. hominis* was lower than Nanjing, where 18.8% had *C. hominis*. In contrast, only one sample from Qingdao was positive for *C. hominis*. In Nanjing and Qingdao, the occurrence of *C. hominis* was probably more common than the data suggested, as *C. muris* and other genotypes from rodents in the wastewater distribution system were the dominant *Cryptosporidium* spp. in samples from the two cities, which could have masked the concurrent presence of *C. hominis*. It is known that minor populations of other species or genotypes in specimens are frequently under-detected with *Cryptosporidium* genus-specific primers [35]. The higher occurrence of *C. muris* in samples from Nanjing and Qingdao could be the result of higher number of rodents in the wastewater distribution system. Therefore, the distribution of *Cryptosporidium* species in these cities may be affected by human populations in the city as well as rodents in the sewer system.

In agreement with this conclusion, other human-pathogenic species that are traditionally associated with zoonotic transmission of cryptosporidiosis were mostly detected in wastewater samples from the four cities at low frequencies. Thus, *C. felis* was not found in any samples, and *C. parvum*, *C. canis*, and *C. cuniculus* were each found in only 2–5 samples. The only exception was *C. meleagridis*, which was found in all four cities studied and in 22 of 210 (10.1%) *Cryptosporidium*-positive wastewater samples (Table 1). The isolated detection of *C. parvum*, *C. canis*, and *C. cuniculus* was probably largely the result of *Cryptosporidium* contamination from domestic animals, as rodents, dogs and rabbits are known hosts of these *Cryptosporidium* species, respectively, in addition to humans. Likewise, pet birds and other domestic birds like chickens and pigeons might have also contributed to the relative high occurrence of *C. meleagridis*, as the more common avian *Cryptosporidium* species, *C. baileyi*, was also found in 15 of the 217 (6.9%) *Cryptosporidium*-positive samples. Nevertheless, the higher occurrence of *C. meleagridis* than *C. baileyi* in wastewater samples indicated that some of the *C. meleagridis* probably originated from humans. In a recent study conducted in in-patients in three hospitals in Shanghai, *C. meleagridis* was detected in 6 of 28 (21.4%) sporadic cases of cryptosporidiosis [36]. Although some of the *C. meleagridis* could have been transmitted anthroponotically, we could not rule out the possibility that some zoonotic transmission of *C. meleagridis* may be occurring in the study areas. Previously, anthroponotic transmission has been identified as a major route in cryptosporidiosis epidemiology in most other developing countries [37].

There could be some differences in the transmission of *C. hominis* among the cities studied, at least between Shanghai and Wuhan. In Shanghai, 29 of the 47 (61.7%) *C. hominis*-positive samples subtyped had three unique subtypes (IbA19G2, IbA20G2, and IbA21G2) of the Ib subtype family. In Wuhan, however, none of the Ib subtypes were found in any of the 10 *C. hominis*-positive samples subtyped. There are not enough *C. hominis*-positive samples from Nanjing and Qingdao to infer *C. hominis* transmission, although IbA19G2 was found in one of the three *C. hominis*-positive samples from Nanjing. Interestingly, the Ie subtype IeA12G3T3 that was previously thought to be unique in Shanghai [16] was found in all other cities studied, and was the dominant *C. hominis* subtype in Wuhan, being found in 9/10 *C. hominis*-positive samples.

Anthroponotic transmission is probably also important in the epidemiology of giardiasis in China. The majority (296 of 319 or 92.8%) of samples genotyped and subtyped had the *G. duodenalis* sub-assemblage A-II. The predominance of sub-assemblage A-II, a parasite mainly found in humans, and the absence of sub-assemblage A-I, a parasite more often found in animals [6], suggests that zoonotic transmission might not play an important role in giardiasis transmission in the study cities. In some samples, several subtypes of assemblage B, which infects humans, ruminants, dogs and other mammals, were also found. Like the sub-assemblage A-II, humans are the likely source for these parasites. This was supported by the absence of animal-specific assemblages of *G. duodenalis*, such as C and D, which are common in dogs, E, which is common in livestock, F, which is common in cats, and G. which is common in rodents [6]. The absence of assemblage G was surprising considering the common presence of rodents in wastewater in cities studied and the high prevalence of *Cryptosporidium* spp. of rodents (Table 1). This was possibly because rodents were more commonly infected with *G. muris* than *G. duodenalis* assemblage G [38], [39]. The primers used in this study are largely *G. duodenalis*-specific, thus have a poor sensitivity in detecting *G. muris*. Only a single target was used in genotyping and subtyping *G. duodenalis*, which could be problematic for assemblage B, as it has been shown to exhibit heterozygosity at some genetic loci.

The transmission of *E. bieneusi* in China is less clear. The majority of *E. bieneusi* found in this study belonged to Group 1 [1], [9]. Parasites in this group infect both humans and animals. Within this group, genotype D was the dominant one, being found in 279/338 (82.5%) *E. bieneusi*-positive samples. The known hosts for genotype D include humans, cattle, pigs, dogs, and some wild mammals. In this study, the contribution of these animals to genotype D was probably limited, as host-adapted *E. bieneusi* genotypes of cattle (BEB6 of group 2), pigs (EbpD of 1e), and wild mammals (groups 3–5) were not found or only found in one sample each (Table 3). The only host-adapted genotypes found in wastewater samples were the called outlier genotype from dogs and two novel genogroups (group 1i and group 6). As they were mostly found in samples from Qingdao, rodents were the likely sources for the two new genogroups. We also cannot rule out the contribution of rodents to genotype D and other minor genotypes in group 1 found in wastewater, as these genotypes generally lack host specificity.

Genotyping data from *Eimeria* spp. and *Cyclospora* spp. generated from wastewater samples mostly support the interpretations on transmission of human-pathogenic protists and attribution of contamination sources. As *Eimeria* spp. and *Cyclospora* form host-specific clusters in phylogenetic analysis of the SSU rRNA sequences, they serve as good markers for contamination source attribution. Thus, the contribution of birds to *C. meleagridis* in wastewater was supported by the occasional finding of *Eimeria* spp. from chickens and other birds. The latter were probably from pet birds, which are known to be infected with *C. meleagridis* in China [40]. Likewise, the common occurrence of *Eimeria* spp. from rodents (seen in 97 samples) was also in agreement with the occurrence of *C. muris* in wastewater samples, especially those from Qingdao and Nanjing. The only discrepancy was the common occurrence of *E. alabamensis* (AF291427) or *E. alabamensis*-like sequences in 167 wastewater samples. Although *E. alabamensis* is a known parasite of cattle, *C. parvum*, *C. andersoni*, *G. duodenalis* assemblage E, and *E. bieneusi* group 2, all of which are common parasites of cattle, were largely absent in wastewater samples in this study. Whether the *E. alabamensis* sequence seen in this study was from cattle is still uncertain, as there are two very different SSU rRNA sequences of *E. alabamensis* in the GenBank (AF291427 and AY876926), with AY876926 forming a cluster with other bovine and ovine *Eimeria* spp. while AF291427 (the type seen in wastewater samples) was alone by itself in a different part of the phylogenetic tree.

In summary, results of this study indicated that molecular surveillance of enteric parasites in wastewater can be used to assess of the characteristics of endemic transmission of pathogens. The data acquired have shown a common occurrence of *Cryptosporidium* spp., *G. duodenalis* and *E. bieneusi* in urban communities in China, although traditional epidemiologic studies are few and routine surveillance of these pathogens is non-existent. These data have also shown a major role of anthroponotic transmission in the epidemiology of cryptosporidiosis, giardiasis, and microsporidiosis in four cities in China. They have also led to the identification of the occurrence of several rare parasites in China, including a new *Cryptosporidium* genotype recently seen in a patient in the United Kingdom [26], the newly identified human pathogen *C. cuniculus*, which was responsible for a recent waterborne outbreak of cryptosporidiosis in the United Kingdom [28], and *C. cayetanensis*, which was only recently identified in humans in China [41], [42]. Thus, genotyping and subtyping enteric parasites in wastewater can be an effective and economical supplement to conventional surveillance and epidemiologic tools. For example, giving the complexity of *C. hominis* population in wastewater in Shanghai [16], the finding of only two major subtypes from *C. hominis* in 86 of 3,245 in-patient in a pediatric hospital in Shanghai has led to the recent identification of a hospital-associated outbreak of cryptosporidiosis [36]. Nevertheless, caution should be exercised when interpreting transmission routes and dynamics using data from molecular surveillance of pathogens in urban wastewater. When some species are shed in larger numbers or longer durations than others, molecular techniques that can detect minor species should be adopted to avoid the misinterpretation of the data. More studies of wastewater samples from other areas are needed to validate the utility of the molecular surveillance system, perhaps in concurrence with case-control or longitudinal studies in humans.
